# GCI‐Based Affinity Screening of Synthetic Oligomannosides toward Concanavalin A

**DOI:** 10.1002/chem.202502137

**Published:** 2025-09-20

**Authors:** Davide Rubes, Sara Tengattini, Massimo Serra, Teodora Bavaro, Caterina Temporini, He Wang, Yongmin Zhang, Francesca Rinaldi, Marco Terreni, Enrica Calleri

**Affiliations:** ^1^ Department of Drug Sciences University of Pavia Via Taramelli 12 27100 Pavia Italy; ^2^ CNRS, Institut Parisien de Chimie Moléculair, UMR 8232 Sorbonne Université 4 Place Jussieu 75005 Paris France

**Keywords:** binding‐affinity, concanavalin a, grating‐coupled interferometry, mannosylation, neo‐glycoproteins

## Abstract

Herein, we report the first application of grating‐coupled interferometry (GCI) to quantify the binding affinity of synthetically defined mannose‐based *neo*‐glycoproteins to Concanavalin A (ConA), a model lectin for carbohydrate recognition. Mono‐, di‐, and tri‐saccharides were conjugated to carrier proteins to create multivalent ligands in a structurally defined yet heterogeneous format. Unlike free sugars, which can interact with lectins in multiple orientations, conjugation constrains sugar presentation and provides a more realistic proxy of how glycans are encountered in glycoconjugates. This approach enabled a systematic comparison of sugar epitopes, confirming that Man(α1,2)Man motif shows stronger binding than Man(α1,6), and that the trisaccharide displays the highest affinity. Notably, the multivalent display of these relatively simple sugar motifs resulted in affinities comparable to or higher than those of natural glycoproteins, highlighting the contribution of valency and presentation rather than glycan complexity. Dissociation constants measured by GCI are consistent with literature values obtained by surface plasmon resonance (SPR) and other methods, confirming the reliability of this platform. This work establishes GCI as a reliable tool for affinity profiling of glycoconjugates and highlights the value of a simple and reproducible screening strategy for evaluating structure–affinity relationships of glycans in a presentation format closer to their intended applications.

## Graphical abstract

This study describes the development of a reliable GCI method for the affinity determination of *neo*‐glycoproteins toward concanavalin A (ConA), a model lectin. The final goal is to investigate multivalent carbohydrate–lectin interactions.

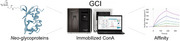

## 1 Introduction

Carbohydrate‐protein interactions have a fundamental role in many physiological and pathological processes.^[^ ^1^, ^2^ ^]^ In particular, mannosylation represents a strategy to target mannose‐binding C type lectin receptors (e.g., MR/CD206, DC‐SIGN/CD209, L‐SIGN/CD209L, Dectin‐2) on antigen presenting cells (APCs). Among these, mannose receptor (MR) is a particularly well‐characterized type I transmembrane protein which can bind a variety of endogenous and pathogen‐associated ligands and participates in antigen cross‐presentation and processing, contributing to both humoral and cellular immune responses.^[^ ^3^ ^]^

Efforts to develop oligosaccharides for MR‐mediated active delivery have focused on identifying optimal sugar epitopes. MR shows affinity for l‐fucose and d‐mannose glycans, with complex branched structures, such as the Man_9_GlcNAc_2_ core, often regarded as the gold standard ligands based on a Man(β1,4)GlcNAc anomeric core. These glycans can be obtained by isolation from natural sources, as a mixture of complex oligosaccharides, or prepared in pure form by means of frequently demanding synthetic procedures.^[^ ^4^ ^]^

Studies on simplified analogues of these ligands reveal contrasting results, with varying disaccharide and trisaccharide affinities and rankings depending on their linkages and assay conditions. Moreover, most studies have been performed with the sugar in solution, which may not accurately reflect its proper orientation and exposure to the receptor when bound to a carrier surface, as occurs in the preparation of glycoconjugate products. Additionally, to achieve detectable binding, affinity evaluations reported in the literature required a relatively high (micromolar) concentration of sugars or sugar derivatives.^[^ ^5^, ^6^, ^7^, ^8^, ^9^ ^]^

Besides ligand structure, the mode of ligand presentation plays a crucial role in binding. This is especially true for carbohydrate‐protein interactions, where binding strength often relies on the formation of multivalent complexes arising from the simultaneous engagement of multiple ligands with several receptors.^[^ ^2^, ^10^ ^]^ Therefore, the design of multivalent ligands allows to increase the affinity to carbohydrate receptors.^[^ ^1^ ^]^

From the binding point of view, surface plasmon resonance (SPR) is considered a valuable technique, since it provides insights into ligand properties, allowing measurement of binding affinities and kinetic data. This technique provides several advantages over other binding assays, including the possibility to perform label‐free and real‐time analyses. It allows for the determination of both affinity constants and kinetic parameters of an interaction, while requiring low amounts of samples for the analysis.

Concanavalin A (ConA) is a widely used lectin model for evaluating carbohydrate interactions with C‐type lectins. Like other C‐type lectins, ConA requires metal ions to mediate carbohydrate binding. These ions mediate the interaction either directly (in the case of animal lectins such as MR) or indirectly (in the case of plant lectins from the Leguminosae family, such as ConA).^[^ ^11^, ^12^, ^13^ ^]^ Beyond subtle differences, recent studies confirm that ConA remains a highly relevant structural and functional model for studying carbohydrate–C‐type lectin interactions.^[^ ^14^ ^]^

Several SPR studies on ConA‐carbohydrates interactions are described in literature. In particular, the binding between glycoproteins,^[^ ^2^, ^15^, ^16^, ^17^ ^]^ glycopolymers,^[^ ^18^, ^19^ ^]^ glycoliposomes,^[^ ^20^ ^]^ glycodendrimers,^[^ ^21^, ^22^ ^]^ glycoconjugated nanoparticles^[^ ^23^ ^]^ or glycan‐coated vesicles^[^ ^24^ ^]^ and ConA was investigated. Other studies have established that monosaccharides bind ConA with relatively weak affinities, typically in the millimolar–micromolar range (10^−3^–10^−^⁶ M).^[^ ^25^, ^26^ ^]^ Increasing glycan complexity can improve binding, as exemplified by Man_9_‐2AB with a Kd of 0.63 µM.^[^ ^26^ ^]^ The most striking affinity enhancement, however, has been consistently associated with multivalent presentation of even simple mannose units, which can lower apparent Kd values to the nanomolar range. Within these multivalent systems, the relative contribution of extending the glycan chain (e.g., from mono‐ to di‐ or trisaccharides) has been reported, but often in heterogeneous formats. This underscores the need for systematic evaluation of short, synthetically defined mannose epitopes displayed on proteins in a controlled and comparable manner—the focus of the present study.

Recently, some improvement of SPR technology have been developed. Among these, grating‐coupled interferometry (GCI) is particularly interesting. This technique allows to study interactions based on the changes in refractive index occurring at a sensor surface, as SPR technology. However, while in SPR a localized evanescent field is created on the sensor chip, in GCI the light runs through the entire length of the sample enhancing the sensitivity and the signal‐to‐noise ratio. More specifically, GCI stands out as one of the most sensitive techniques among label‐free biosensors. Thanks to its features, GCI allows a rapid and accurate measurement of strong and weak binders in a wide affinity range.^[^ ^27^, ^28^ ^]^

Over the past years, we have developed a chemoenzymatic method enabling the rapid synthesis of linear and branched sugars using acetyl as the sole protecting group. This approach is based on the preparation of fully acetylated building blocks bearing a free hydroxyl group at the desired position via enzymatic regioselective hydrolysis,^[^ ^29^, ^30^ ^]^ which can then be assembled to produce glycans with different anomeric linkers,^[^ ^30^, ^31^ ^]^ thereby avoiding complex chemical procedures.

Accordingly, in this work, mannose glycans with an anomeric thiocyanomethyl group were synthesized and conjugated with two nonimmunogenic proteins, ribonuclease A (RNase A) and human serum albumin (HSA) to generate multivalent ligands. The affinity between the *neo*‐glycoproteins and ConA was analyzed by GCI as a means to assess glycan presentation in a conjugated format, rather than in solution. To our knowledge, this is the first time GCI has been exploited to this aim. In particular, we focused on di‐ and trisaccharide derivatives with different linkages, which enable systematic comparison of short mannose epitopes when displayed on proteins, complementing earlier studies that primarily examined complex natural glycans or monosaccharide conjugates.

To our knowledge, this represents the first application of GCI to such constructs. Our approach is designed to provide systematic insights into how linkage and chain length influence C‐type lectins binding, while highlighting the contribution of multivalency and presentation rather than glycan complexity alone.

This work addresses the longstanding challenge of correlating glycan structure with receptor binding in a controlled format that better reflects glycan display in conjugates compared to free sugars in solution and introduces a powerful analytical platform for glycan–receptor studies for the design of multivalent glycoconjugate with well‐defined structures and enhanced functional performance.

## 2 Results and Discussion

### 2.1 Synthesis of IME Saccharides and Neo‐Glycoproteins

**Scheme 1:**
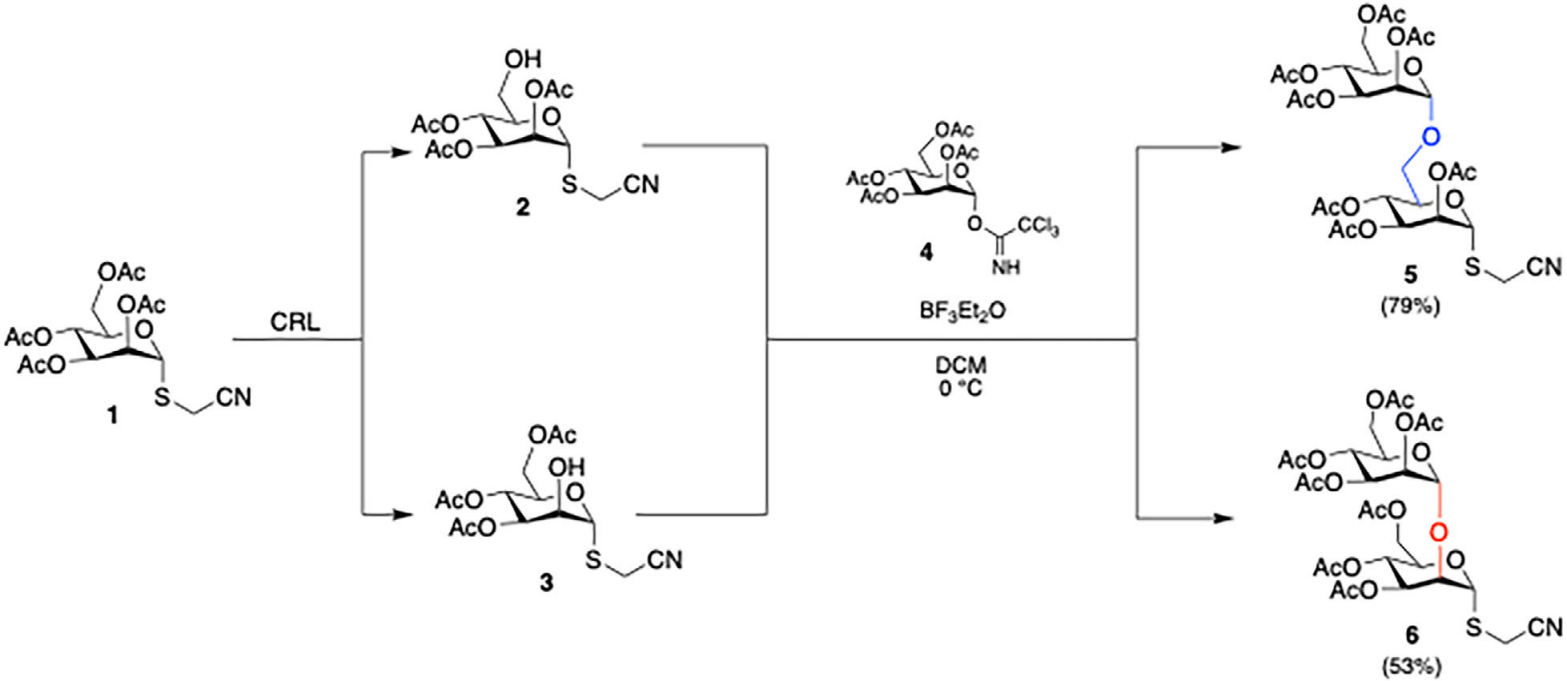
Synthesis of Man(α1,6)Man‐ and Man(α1,2)Man‐thiocyanomethyl derivatives **5** and **6**.

The thiocyanomethyl disaccharides **5** ^[^ ^32^ ^]^ and **6** ^[^ ^31^ ^]^ were prepared by following a synthetic strategy previously developed by our group (Scheme 1).^[^ ^33^ ^]^ One of the key steps was the preparation of the functionalized monosaccharide **1**,^[^ ^34^ ^]^ which was performed through a synthetic sequence involving anomeric chlorination of peracetylated d‐mannose, the subsequent treatment with thiourea to replace the chlorine atom with the thiopseudourea moiety, and the final conversion into the desired thiocyanomethyl group by using sodium bisulfite, potassium carbonate, and chloroacetonitrile.

The C6‐, and C2‐deacetylated derivatives **2** and **3** were obtained in 50% and 40% recovered yield, respectively, following enzymatic hydrolysis of **1** in the presence of candida rugosa lipase (CRL). In this case, the low enzyme selectivity between C2 and C6 positions proved to be an added value, because it provided fast access to both building blocks required for the preparation of disaccharides **5** and **6**. Moreover, the enzymatic hydrolysis of the peracetylated precursor allowed the synthesis of the desired glycans only using the acetyl group as a protecting group avoiding the tedious development of a classic chemical approach, that requires the use of different protecting groups and a quite high number of synthetic steps.

In fact, the second building block used, was the trichloroacetimidate (TCA)‐activated mannose **4**,^[^ ^35^ ^]^ prepared in a few steps starting from commercial d‐mannopyranose. This last derivative was firstly converted into the corresponding peracetylated analogue, and then selectively deprotected at the anomeric position and activated with trichloroacetonitrile and diazabicycloundecene (DBU) to give **4**. Afterwards, the glycosyl donor **4** was reacted with C6‐deprotected sugar **2** in the presence of boron trifluoride diethyl etherate (BF_3_Et_2_O) to gain Man(α1,6)Man disaccharide **5** in 79% yield. The application of the same reaction conditions allowed the condensation between **4** and C2‐deprotected derivative **3** to takes place, affording Man(α1,2)Man disaccharide **6** in 53% yield.

**Scheme 2:**
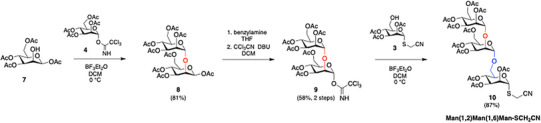
Synthesis of the thiocyanomethyl trisaccharide.

Similarly, the synthesis of previously unreported thiocyanomethyl trisaccharide **10** entailed an initial condensation between **4** and C2‐deprotected tetraacetyl mannopyranose **7** (Scheme 2).^[^ ^36^ ^]^

Also in this case, the use of BF_3_Et_2_O in DCM at 0 °C furnished the corresponding disaccharide **8** (y: 81%),^[^ ^37^ ^]^ which was treated with benzylamine to deprotect the anomeric position and then activated with trichloroacetonitrile.^[^ ^38^ ^]^ The obtained TCA‐derivative **9**, once reacted in the presence of thiocyanomethyl building block **3**, allowed the isolation of desired Man(α1,2)Man(α1,6)Man trisaccharide **10** in 87% yield. The α‐anomeric configuration was confirmed by an undecoupled ^13^C NMR experiment, revealing ^1^J_C1,H1_ of ∼170 Hz, in agreement with values reported for α‐glycosidic linkages^[^ ^39^ ^]^ (see Supporting Information, Figure S7).

**Scheme 3:**
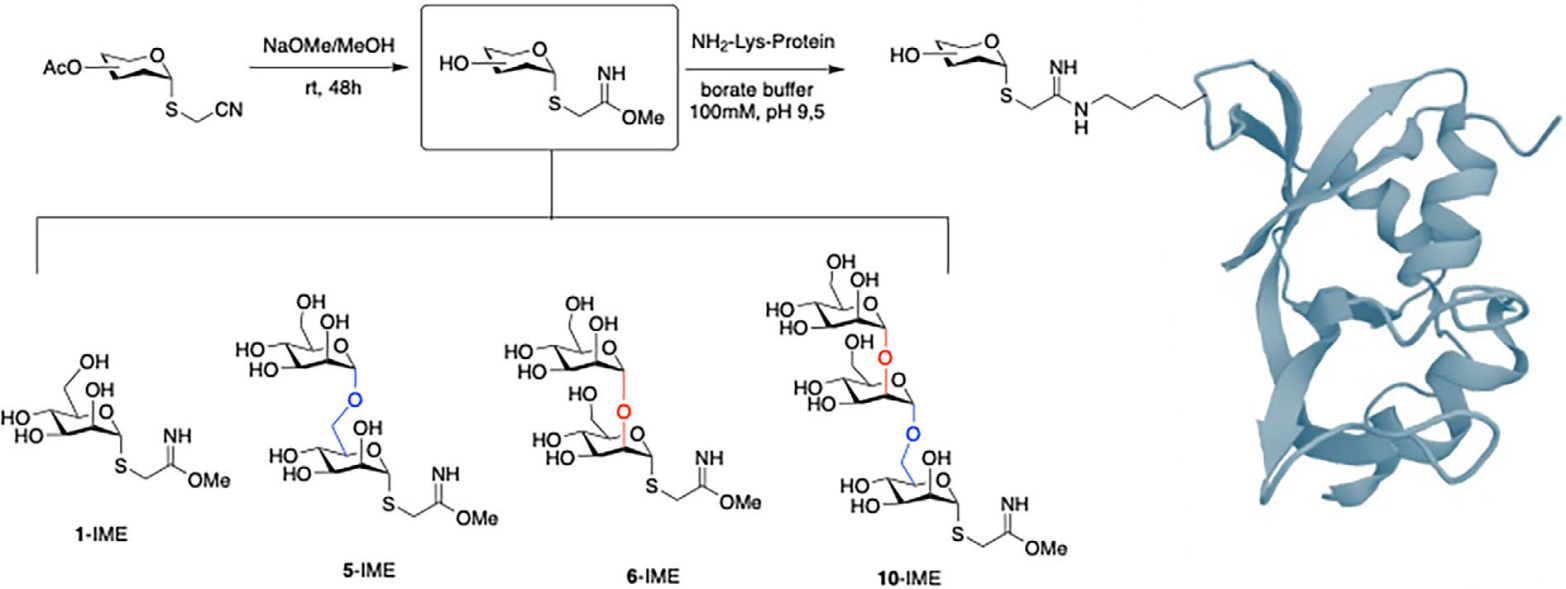
Synthesis of iminometoxyethyl (IME)‐activated sugars and their use in the preparation of *neo*‐glycoproteins.

All the glycans have been prepared using the acetyl as the only protecting group. This makes it possible to obtain one pot final deprotection and activation of the thiocyanomethyl group. Thus, before being used in the glycosylation step, the thiocyanomethyl sugars **1**, **5**, **6**, and **10** were converted into the corresponding iminometoxyethyl derivatives **1‐**IME, **5‐**IME, **6‐**IME, and **10‐**IME through treatment with sodium methoxide in methanol (Scheme 3).

The glycosylation of proteins using IME‐glycans selectively targets lysine residues. Glycosylation of RNAse A and HSA has been carried out by adapting our previously reported method.^[^ ^32^, ^40^ ^]^ The proteins were dissolved in sodium tetraborate buffer and incubated with IME‐activated sugars at room temperature. After 24 hours, the glycosylation degree was evaluated using mass spectrometry (MS, Figures S1, S2). The resulting glycoproteins are heterogeneous constructs, composed of a mixture of proteins carrying a variable number of glycans on different lysines; the average glycan loading on each carrier protein is reported in Table 1. The glycosylated proteins were then purified, quantified, and submitted to GCI analysis.

**Table 1:** Average sugar/protein ratio defined by HILIC‐UV‐MS or MALDI‐ToF, association rate constant (ka), dissociation rate constant (kd), and equilibrium dissociation constant (Kd) derived from the kinetic analysis of the glycoconjugates. For RNase B and RNase A‐Man, average values, and associated standard deviations derive from 3 replicate analyses on 3 different sensor chips. Errors associated to ka and kd for all *neo*‐glycoproteins can be found in Figure S4.

Entry	Protein	Sugar	Average glycan loading [mol/mol]	ka [M^−1^s^−1^]	kd [s^−1^]	Kd [nM]
1	RNaseB	GlcNAc_2_‐Man_5‐9_	1.0	5.50 ± 0.85 *10^4^	2.41 ± 0.21 *10^−3^	44.319 ± 6.527
2	RNaseA	Man (**1**)	5.7	3.81 ± 0.28 *10^4^	1.72 ± 0.29 *10^−3^	44.964 ± 5.609
3	RNaseA	Man(α1,6)Man (**5**)	7.5	8.19*10^4^	1.82*10^−3^	22.196
4	RNaseA	Man(α1,2)Man (**6**)	7.6	1.07*10^5^	1.73*10^−3^	16.181
5	RNaseA	Man(α1,6)Man(α1,2)Man (**10**)	7.9	2.12*10^5^	8.75*10^−4^	4.132
6	HSA	Man(α1,6)Man (**5**)	15.3	4.13*10^4^	1.66*10^−3^	40.128
7	HSA	Man(α1,2)Man (**6**)	23.5	1.02*10^5^	7.53*10^−4^	7.369
8	HSA	Man(α1,6)Man(α1,2)Man (**10**)	16.4	8.58*10^4^	3.70*10^−4^	4.311

### 2.2 GCI Method Development

ConA was selected as a model lectin with the aim of studying the interactions of the different sugars with the receptor. ConA is a plant lectin refined from jack beans. It can exist as a dimer (pH 5.5) or as a tetramer (pH > 7.0) and the molecular weight of the monomer is 25.5 kDa. It is reported to possess different isoelectric points ranging from 4.5 to 5.5, corresponding to different isoforms.^[^ ^41^ ^]^

For ConA immobilization, amine coupling chemistry was selected based on studies describing SPR experiments with this lectin. In these works, a covalent immobilization on polycarboxylated sensor chips to obtain high‐density lectin surfaces was described.^[^ ^21^, ^22^ ^]^ Coherently, in the present work, ConA was covalently immobilized by amine coupling on a 4PCH sensor chip functionalized with a high capacity polycarboxylate layer. To select the best immobilization conditions for amine coupling, a pH scouting study was performed. The tested pHs (4.0, 4.5, and 5.0) were chosen based on the isoelectric points of ConA. In fact, the induction of a positive charge on the protein (pH < isoelectric point) is known to promote electrostatic interactions between the protein and the negatively charged surface allowing protein preconcentration before immobilization.^[^ ^42^ ^]^ The best results in term of immobilization yield and protein stability were obtained with pH 4.5, which was therefore selected for the immobilization. The detailed immobilization procedure is described in Materials and Methods Section and allowed to obtain an immobilization yield of 8237 ± 889 pg/mm^2^ (mean of 3 determinations).

**Figure 1:**
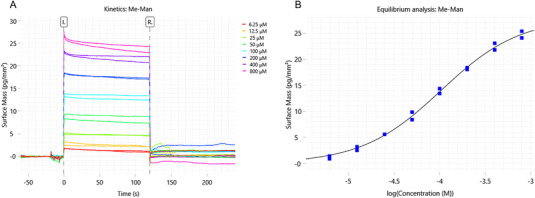
A) Kinetic and B) equilibrium analysis of Me‐Man, with sensorgrams showing fast on‐ and off‐rates.

ConA proper immobilization was assessed by performing a kinetic analysis in the presence of methyl α‐d‐mannopyranoside (Me‐Man) as a reference compound. Following an SPR method already described,^[^ ^21^ ^]^ Me‐Man was analyzed in a concentration range of 6.25–800 µM as detailed in the Materials and Methods Section. As reported in literature,^[^ ^21^, ^22^ ^]^ due to the fast on‐ and off‐rates of the sensorgrams (see figure 1A), it was not possible to apply a kinetic evaluation and an equilibrium analysis was performed for data processing (Figure 1). The 1:1 interaction fitting model resulted in a measured dissociation constant (Kd) of 92 ± 11 µM (mean of 3 determinations). The Kd is coherent with literature data obtained by SPR,^[^ ^21^, ^22^ ^]^ confirming the proper immobilization of ConA and the comparability between SPR and GCI results. Noteworthy, data obtained on 3 different chips were consistent with each other.

The ConA chips were then tested by the analysis of a model glycoprotein, RNase B. This molecule has a single glycosylation site but exists in different glycoforms, all of which share a chitobiose core, a disaccharide made up of two N‐acetylglucosamine (GlcNAc) units linked by a β(1→4) bond. This core is further connected to varying numbers of mannose residues, ranging from 5 to 9, thus including the gold standard ligand GlcNAc_2_Man_9_ (Figure S3).

The different glycoforms are reported to have Kds ranging from 20 to 360 nM toward ConA.^[^ ^43^ ^]^ Therefore, a wide concentration range (2 nM–5 µM) was tested in the first kinetic analyses. However, a saturation of ConA was observed, especially at the highest concentrations, requiring a reduction of RNase B concentrations and the addition of a regeneration step after each sample injection.

**Figure 2:**
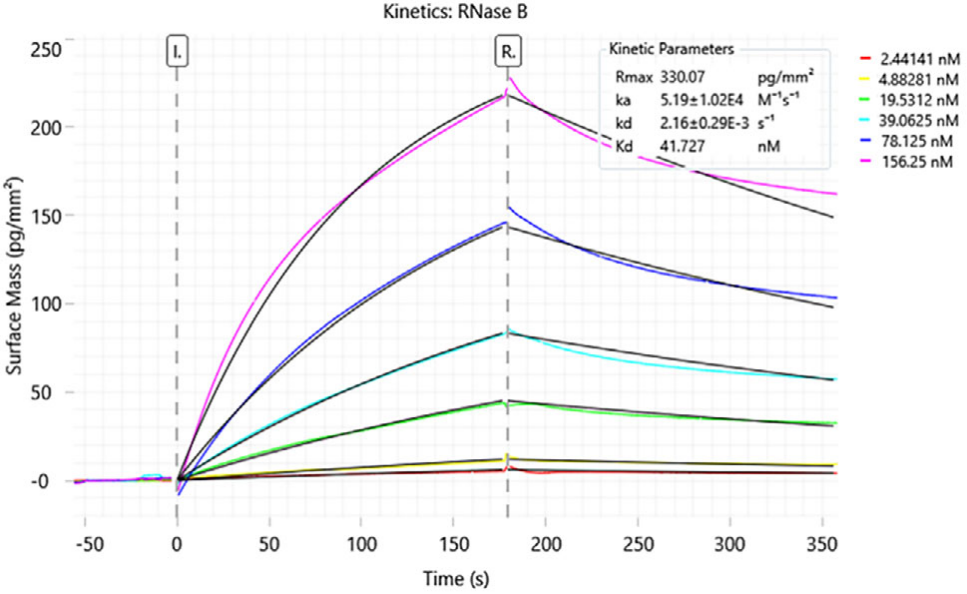
GCI kinetic analysis of RNase B.

To identify suitable regeneration conditions, various solutions were screened. A 50 mM solution of Me‐Man was tested due to its affinity and fast dissociation from ConA. Additionally, acid (100 mM glycine, pH 1.7) and basic (50 mM glycine, pH 9.5) solutions were evaluated for their ability to disrupt the electrostatic interactions. Furthermore, the running buffer without calcium and magnesium ions, both of which induce conformational changes essential for carbohydrate binding to ConA,^[^ ^12^ ^]^ was injected as a potential regeneration solution. Based on the results, the 50 mM Me‐Man solution was selected for the regeneration since, due to the mild conditions, it allowed to displace the bound RNase B without affecting the ConA stability. The addition of one injection of 50 mM Me‐Man and one injection of running buffer (blank) after each sample concentration allowed to obtain good sensorgrams and reliable kinetic parameters (Figure 2). However, the consecutive analysis of several glycoproteins pointed out the need for a stronger regeneration solution between samples. Therefore, solutions of 300 mM Me‐Man and 1 M NaCl, injected separately or in a mixture, were also investigated. The two separate solutions proved to be effective for the intended aim.

Different association and dissociation times were also evaluated. The best conditions for kinetic analyses were found to be 10 µL/minute flow rate, 180 s association and dissociation time, 2–312 nM concentration range, regeneration with 50 mM Me‐Man after each glycoprotein injection and with 300 mM Me‐Man and 1 M NaCl after each analysis. By applying these conditions, a Kd of 44 ± 6 nM (mean of 3 determinations) was obtained for RNase B. As already observed for Me‐Man, comparable Kd values were measured on 3 different sensor chips, confirming the repeatability of the immobilization and analysis procedures.

### 2.3 GCI Affinity Determination of *Neo*‐Glycoproteins

The developed GCI method was then applied to the analysis of *neo*‐glycoproteins prepared as previously described. The affinity to ConA was measured after conjugation of IME‐glycans with two nonimmunogenic carrier proteins characterized by a different molecular weight and a different number of glycosylation sites: RNase A and HSA. RNase A (MW = 13.7 Da) shares the same amino acid sequence as RNase B, but it is naturally nonglycosylated, and contains 10 lysines as potential glycosylation sites. In contrast, HSA (Mw = 66.4 kDa), the main transport protein in human blood, features 60 Lys in its amino acid sequence.

RNase A, glycosylated with monosaccharide **1**‐IME was selected as a model *neo*‐glycoprotein to assess the repeatability of the results, analyzing its affinity toward ConA on 3 different chips, similarly to Me‐Man and RNase B. A Kd value of 45 ± 6 nM was obtained, confirming once again the reliability of the analytical procedure. Notably, the measured affinity is comparable to the one of RNase B, which contains more complex sugars in its structure but has only a single glycosylation site. In contrast, the average sugar/protein ratio of RNase A‐Man defined by MS analysis is 5.7 mol/mol. This result suggests that the binding of multiple sugars to the RNase A surface has a positive effect on the interaction with ConA, in agreement with the principle of multivalency.

The objective of this study is to compare the in‐house synthesized short‐chain mannans to identify the minimal sugar epitope interacting with ConA in multivalent constructs. Two disaccharides characterized by different glycosidic linkages, Man(α1,6)Man (**5**), and Man(α1,2)Man (**6**), were first compared. The (α1,2) disaccharide revealed a better interaction with ConA compared to Man(α1,6)Man using both carrier proteins (Table 1 and Figure S4). The glycan loading of the two disaccharides on RNase A was comparable (7.5,7.6, Table 1), confirming that the observed difference in the dissociation constant is related to the type of sugar. Instead, for HSA the higher number of Man(α1,2)Man per protein could play a role in the more marked difference in Kd (Table 1). However, it should be noted that the average sugar/protein ratio of Man(α1,2)Man on HSA is 1.5 times higher than the one of Man(α1,6)Man, while the Kd is 5.4 times lower. Therefore, it could be postulated that the increased affinity is mainly due to the type of disaccharide rather than its loading on the protein. This observation is in accordance with structural studies indicating that sugars including the Man(α1,2)Man motif show an higher affinity for the lectin MR.^[^ ^13^, ^44^ ^]^

Given the stronger recognition displayed by the α 1,2 motif, the activated trisaccharide **10**‐IME, endowed with this glycosidic bond pattern, was synthesized and conjugated to RNase A and HSA carrier proteins. A trend of increasing affinity at increasing sugar chain lengths was observed, with the trisaccharide derivative showing the best interaction with ConA (Table 1 and Figure S4). These results are in agreement with a recently published study in which authors observed that, among different mammalian sugars, MR mainly bound to mannose‐terminating glycans, with the mannose trisaccharide being one of the most prevalent variants.^[^ ^45^ ^]^ For this glycan, conjugation with RNase A resulted in a slightly higher average sugar‐to‐protein ratio compared to the disaccharide derivative. However, similar to what was observed for HSA conjugated with disaccharides, the marked difference in dissociation constants suggests that the type of sugar present plays a predominant role in determining the affinity of the *neo*‐glycoproteins for the receptor, rather than the sugar loading itself. The smaller affinity difference observed between the HSA *neo*‐glycoproteins Man(α1,2)Man and Man(α1,6)Man(α1,2)Man, compared to those using RNase A as the carrier protein, can be partially attributed to the greater disparity in glycan loading. Remarkably, a marked difference was observed in the dissociation constant of the trisaccharide conjugated to both carrier proteins compared to the (α1,6) disaccharide, further confirming the higher affinity of the Man(α1,2)Man motif for ConA.

Nonglycosylated RNase A and HSA were used as negative controls and their interaction with ConA was found to be absent or negligible (Figure S5), proving that the observed affinity is related only to the sugar moieties of the glycoconjugates.

### 2.4 Comparison with Other Studies

To place our findings in context, we compared the GCI‐derived affinities of our *neo*‐glycoproteins with literature values for a range of multivalent carbohydrate displays interacting with ConA (Table 2). As expected, multivalency provides a dramatic enhancement over monovalent sugars in solution,^[^ ^25^, ^26^ ^]^ and our dissociation constants (Entries A) fall within the same nanomolar range reported for glycopolymers, glycodendrimers, and other *neo*‐glycoproteins studied by SPR or glycan arrays (Entries B–F). This cross‐validation highlights that GCI provides results consistent with established biosensor methods, while offering high sensitivity and excellent signal‐to‐noise ratios.

**Table 2:** Comparison of the Kd values obtained in the present work with affinity literature data on multivalent carbohydrate–lectin interactions.

Entry	Ligand / format	Method	Reported value [Kd or Ki or IC50)]
A (This work)	RNaseA‐Man, RNaseA‐Man(α1,6)Man, Man(α1,2)Man, Man(α1,6)Man(α1,2)Man; HSA analogues	GCI	RNaseA‐Man: 45 ± 6 nM; Man(α1,6)Man: 22 nM; Man(α1,2)Man: 16 nM; Man(α1,6)Man(α1,2)Man: 4 nM; HSA conjugates: 40 / 7.4 / 4.3 nM
B^[^ ^2^ ^]^	Man_6_‐BSA, BSA‐Manα *neo*‐glycoproteins	Glycan microarray	Man_6_‐BSA: 49 ± 29 nM; BSA‐Manα: 69 ± 7 nM (apparent Kd)
C^[^ ^43^ ^]^	RNase B (natural glycoforms Man_5_–Man_9_)	SPR	20–360 nM depending on glycoform/assay
D^[^ ^17^ ^]^	Mannan‐BSA *neo*‐glycoproteins	SPR	325–532 nM (depending on mannose density)
E^[^ ^19^, ^21^ ^]^	Mannose glycodendrimers / glycopolymers	SPR	2–20 nM, depending on valency/density. Ki up to 40 times lower for glycopolymers than for sugar in solution; increasing valency reaches plateau (no dramatic increase in potency for glycopolymers longer than the 25mer).
F^[^ ^24^ ^]^	Vesicles	Microplate inhibition assay	Man_9_Asn IC50 1.06 µM Man_9_‐glycan‐coated vesicles (gCVs) IC50 0.14 µM

Within this framework, our systematic comparison of mono‐, di‐, and trisaccharide conjugates (Entries A) highlights clear structure–activity relationships. Consistent with previous reports, the Man(α1,2)Man disaccharide enhances binding relative to the Man(α1,6)Man analogue,^[^ ^13^, ^44^ ^]^ and incorporation of a trisaccharide motif provides an additional, though more modest, gain.^[^ ^45^ ^]^ Overall, defined di‐ and trisaccharide motifs, such as Man(α1,2)Man or Man(α1,2)Man(α1,6)Man, confer extra affinity compared to monosaccharide‐conjugated proteins, although the increase is less pronounced than that observed when comparing simple and more complex sugars in solution.

A particularly noteworthy observation emerges when comparing our short, synthetically defined oligomannosides with systems that employ larger, naturally derived glycans. Several studies have reported multivalent displays of Man_6_ or Man_9_ epitopes, or even heterogeneous mannans (Entries B, D, F). Despite the higher intrinsic complexity of these ligands, the reported affinities are not superior to those obtained with our simple di‐ and trisaccharide conjugates. This reinforces the view that, once presented in a multivalent fashion, the specific glycan size and branching contribute less to binding strength than the overall display format.

Taken together, these observations demonstrate that GCI‐derived affinities reproduce established trends in lectin–glycan recognition and confirm that short, well‐defined oligomannosides, when presented multivalently on a protein scaffold, can achieve binding performances comparable to or exceeding those of natural complex glycans.

## 3 Conclusion

In this study, we exploited GCI to analyze *neo*‐glycoproteins carrying synthetically defined mannose oligosaccharides in a multivalent display format. Although ConA interactions with natural glycoproteins, mannan–BSA conjugates, and monosaccharide derivatives have been reported, systematic comparisons involving defined di‐ and trisaccharides within a neo‐glycoprotein framework are still lacking. Importantly, our GCI‐derived dissociation constants (4–45 nM, depending on glycan motif and carrier protein) are within the same range as those reported by SPR and microarray studies for comparable neo‐glycoproteins and multivalent displays (Table 2). This cross‐validation highlights that GCI provides results consistent with established biosensor methods, while offering high sensitivity and excellent signal‐to‐noise ratios. Our findings that Man(α1,2)Man displays stronger binding than Man(α1,6)Man, and that the trisaccharide motif further enhances affinity, are consistent with structural and array‐based studies reporting preferential recognition of Man(α1,2)‐terminated epitopes. These findings underscore that glycan linkage and presentation, more than size or structural complexity, are key determinants of lectin affinity.

## 4 Materials and Methods

### 4.1 Chemical and Reagents

HEPES, magnesium chloride (MgCl_2_), Tween 20, Concanavalin A (ConA) from Canavalia ensiformis (Jack bean), methyl‐α‐D‐mannopyranoside (Me‐Man), ribonuclease A (RNase A) from bovine pancreas and ribonuclease B (RNase B) from bovine pancreas were from Sigma‐Aldrich (Milan, Italy).

HSA was from Oryzogen (Wuhan, China).

Sodium chloride (NaCl) was purchased from PanReac AppliChem ITW Reagents (Cinisello Balsamo, Italy).

Calcium chloride (CaCl_2_) was from Carlo Erba (Cornaredo, Italy).

N‐hydroxysuccinimide (NHS), N‐(3‐dimethylaminopropyl)‐N′‐ethylcarbodiimide hydrochloride (EDC) and ethanolamine were from XanTec bioanalytics GmbH (Duesseldorf, Germany).

Deionized water was obtained from a Milli‐Q Integral purification system from Merck KGaA (Darmstadt, Germany).

For the synthesis of sugar derivatives, reactants, and chemicals were obtained from commercial suppliers (Sigma‐Aldrich, Burlington, MA, USA (Merck KgaA group), Alfa Aesar Ward Hill, MA, USA (Thermo Fisher Scientific group)), and were used directly without any additional purification. The guidelines described in *Purification of Laboratory Chemicals* ^[^ ^46^ ^]^ were followed for the purification of solvents, which were freshly distilled from the appropriate drying agent. THF was distilled from sodium/benzophenone ketyl, and DCM from CaH_2_. When anhydrous conditions were needed, reactions were performed under N_2_. Flash chromatography using Silica Gel high‐purity grade, pore size 60 Å 70–230 mesh, 63–200 µm (Sigma‐Aldrich) was employed for compound purification. Analytical thin layer chromatography (TLC) was carried out on silica gel F254 precoated aluminum sheets (0.2 mm layer, Merck, Darmstadt, Germany). The plates were visualized using a 254 nm UV lamp, then treated with a 5% H_2_SO_4_ solution in ethanol and heated at 150 °C. The purified compounds were characterized by NMR spectroscopy using a Bruker Advance III 400 MHz spectrometer (Bruker Corporation, Billerica, MA, USA). High resolution mass spectra were acquired by a X500B QTOF System (SCIEX, Framingham, MA 0 1701, USA) equipped with a Twin Sprayer ESI probe and coupled to an ExionLC system (SCIEX). Yields were calculated for compounds purified by flash chromatography and judged homogeneous by thin‐layer chromatography, NMR, and mass spectrometry.

### 4.2 Synthesis of Man(α1,2)Man(α1,6)Man‐SCH_2_CN 10

2′,3′,4′,6′‐Tetra‐O‐acetyl‐α‐D‐mannopyranosyl‐(1→2)‐3,4,6‐tri‐O‐acetyl‐α‐D‐mannopyranosyl trichloracetimidate **9** (60 mg, 0.077 mmol, 1 eq.) and Thiocyanomethyl‐2,3,4‐tri‐O‐acetyl‐α‐D‐mannopyranoside **3** (36 mg, 0.100 mmol, 1.3 eq.) were dissolved in anhydrous dichloromethane (770 µL, 0.1 M) in the presence of activated molecular sieves. The mixture was cooled to −20 °C under nitrogen atmosphere. BF_3_Et_2_O (15 µL, 0.116 mmol, 1.5 eq.) was added and the mixture was stirred for 10 minutes. The reaction was quenched with triethylamine (16 µl, 0.116 mmol, 1.5 eq.), filtered and concentrated in vacuo. The reaction was monitored by TLC (ethyl acetate/cyclohexane 6:4 Rf = 0.10) and purified by flash chromatography (acetone/dichloromethane 1:9) to obtain **10** as a white solid (y: 87%). ^1^H‐NMR (400 MHz, CDCl_3_): δ = 1.98 (s, 3H), 1.99 (s, 3H), 2.07 (2 signals: s, 3H + s, 3H), 2.05 (s, 3H), 2.06 (s, 3H), 2.07 (s, 3H), 2.13 (2 signals: s, 3H + s, 3H), 2.17 (s, 3H), 3.37 (d, *J* = 17.3 Hz, 1H), 3.51 (d, *J* = 17.3 Hz, 1H), 3.54 (dd, *J* = 2.3, 11.0 Hz, 1H), 3.85 (dd, *J* = 6.7, 10.6 Hz, 1H), 3.94 (dt, *J* = 3.0, 10.0 Hz, 1H), 4.01 (t, *J* = 2.4 Hz, 1H), 4.10–4.22 (m, 5H), 4.28 (ddd, *J* = 2.3, 6.9, 9.3 Hz, 1H), 4.89 (d, *J* = 1.8 Hz, 1H), 4.98 (d, *J* = 1.7 Hz, 1H), 5.18–5.40 (m, 8H), 5.46 (d, *J* = 1.1 Hz, 1H); ^13^C{1H} NMR (CDCl_3_, 100 MHz) δ 15.4 (t), 20.5, 20.6, 20.7 (3 signals, 4C + 2C + 1C), 20.9, 62.0 (t), 62.4 (t), 65.6, 66.1 (t), 66.3, 66.4, 68.3, 68.8, 69.1, 69.2, 69.4, 69.7, 70.0, 70.4, 77.1, 81.6, 97.8, 99.3, 115.7 (s), 169.4 (s), 169.5 (s), 169.6 (s), 169.7 (3 signals: 1C, s + 1C, s + 1C, s), 169.9 (s), 170.4 (s), 170.5 (s), 170.8 (s); HRMS (ESI) calculated for C_40_H_53_NNaO_25_S [M + Na]^+^ 1002.2519, found 1002.2505 (Δ = −1.5 ppm) (Figure S6).

### 4.3 General Procedure for Activation to IME Saccharides

Compounds **5**, **6**, and **10** were treated with 2 equivalents of sodium methoxide in methanol (0.05 M) at room temperature for 48 hours. After this time, the reactions were monitored by mass spectrometry. The conversion yield was determined using electrospray ionization mass spectrometry (ESI‐MS) by calculating the ratio of the peak intensity of the activated form to the total intensity of all the ions observed in the mass spectrum (**5** IME yield: 85%, **6** IME yield: 81%, and **10** IME yield: 63%).

### 4.4 General Procedure for Protein Glycosylation

The proteins were dissolved in 100 mM sodium tetraborate buffer (pH 9.5) to reach final concentrations of 4 mg/mL for RNAse A and 10 mg/mL for HSA. The solutions were then mixed with IME‐glycosides at an IME‐glycoside/protein molar ratio of 100:1 and the reaction mixtures stirred at room temperature overnight.

### 4.5 Glycoconjugate Purification by Ultracentrifugation

Glycosylation mixtures were subjected to ultrafiltration to remove excess saccharides and transfer the glycoconjugates into PBS buffer. Amicon Ultra‐Centrifugal Filters (Merck Millipore, Darmstadt, Germany) with a molecular weight cut‐off (MWCO) of 3 kDa for RNase A and 10 kDa for HSA, with a sample volume of 0.5 mL, were used in combination with a centrifuge 5804‐R (Eppendorf s.r.l., Milan, Italy). The filters were preconditioned with PBS (4 °C, 13000 RCF for 10 minutes) before sample loading. The samples were then subjected to two buffer exchange steps (4 °C, 13,000 rpm for 10 minutes) using PBS as the solvent. Sample recovery from the filters was performed at 4 °C, 1000 RCF for 2 minutes.

### 4.6 Glycan Loading Assessment

#### 4.6.1 Hydrophilic Interaction Liquid Chromatography (HILIC)‐UV‐ESI‐MS Analysis

RNase B and glycoconjugates were analyzed by HILIC, a chromatographic mode in which retention increases with sugar loading.

For the LC separation a Dionex UltiMate 3000 HPLC (Thermo Scientific, San Jose, CA, USA) equipped with autosampler, ternary pumps, UV detector, and coupled to a LTQ ion trap mass spectrometer with an electrospray ionization (ESI) ion source (Thermo Finnigan, San Jose, CA, USA) was used. The system's control interface was X‐Calibur software version 2.0.7. Chromatographic conditions were optimized starting from^[^ ^47^ ^]^ for RNAse A glycoconjugates and from^[^ ^30^ ^]^ for HSA glycoconjugates. The HILIC column was an AdvanceBio Glycan Mapping column (150 × 2.1 mm, 2.7 µm) from Agilent Technologies (Palo Alto, CA, USA). The mobile phase consisted of ACN (A) and water (B) both containing 0.1% TFA. For RNase B elution was set as follows: from 20 to 30% B in 1 minute, from 30 to 45%B in 15 minutes. For RNase A glycoconjugates elution was set as follows: from 20 to 30% B in 1 minute, from 30 to 60%B in 15 minutes. For HSA glycoconjugates elution was set as follows: from 20 to 30% B in 1 minute, from 30 to 40%B in 15 minutes.

Samples were analyzed at a concentration of 1 mg/mL. The injection volume was fixed at 2 µL, using the microliter pick‐up function and ACN as the transport liquid. Column temperature was set at 50 °C, flow rate at 0.2 mL/minute and UV absorbance was monitored at 214 nm.

The following MS parameters were applied: positive ion mode, scan range 600–200 *m*/*z* in full‐scan mode, source voltage 4.6 kV, capillary voltage 43 V, sheath gas flow rate 15 (arbitrary units), auxiliary gas flow rate 10 (arbitrary units), capillary temperature 250 °C, tube lens voltage 165 V.

For all the synthetized glycoconjugates, HILIC‐UV‐MS analysis allowed to assess the success of the conjugation by the disappearance of the peak relative to the unmodified protein and the formation of more retained peaks.

For RNase A glycoconjugates, the ESI‐MS spectra were used to define sugar loading. The average glycan loading (mol/mol) for each glycoconjugate preparation was determined by multiplying the percentage relative abundance of each glycoform in the deconvoluted spectrum by the number of incorporated sugar units.

For HSA glycoconjugates, the higher MW of the protein and the heterogenicity of the resulting glycoconjugates required additional analysis by MALDI‐ToF to determine the loading.

#### 4.6.2 MALDI‐ToF Analysis

In order to define the glycan loading for HSA glycoconjugates, the analysis was performed on a MALDI‐ToF (Ultraflex, Bruker) in linear positive ion mode. α‐cyano‐4‐hydroxycinnamic acid was used as the matrix (10 mg/mL in H2O/Acetonitrile (ACN)/0.1% trifluoroacetic acid (TFA)) with dried droplet preparation. External mass calibration was achieved with standard proteins (Promix 3, LaserBioLabs).

The average glycan loading (mol/mol) for each HSA glycoconjugate preparation was determined by calculating the mass shift between the average mass of the glycoconjugate and the unmodified protein, and dividing the mass shift by the molecular mass of each sugar unit.

### 4.7 Glycoconjugate Quantification by Reverse Phase (RP)‐LC‐UV

RP‐LC analysis was performed on an Agilent 1200 Series (Agilent Technologies, Palo Alto, CA, USA) controlled by a ChemStation software (revision B.04.03‐SP2) using a mobile phase composed of water (A) and ACN (B) both containing 0.1% formic acid. The column was an Advance Bio RP‐mAb C4 (2.1×50 mm, 3.5 µm) from Agilent Technologies (Palo Alto, CA, USA). Linear gradient elution was applied for derivatives from both proteins: from 5% to 60% B in 10 minutes for RNase A glycoconjugates and from 20 to 70% B in 10 minutes for HSA glycoconjugates. Injection volume, column temperature, and UV wavelength were set at 2 µL, 70 °C and 214 nm, respectively. A calibration curve was built for each analyte using the unmodified (nonglycosylated) proteins, as glycans do not alter protein UV absorbance. Five concentration levels were considered (from 0.25 to 1.5 mg/mL, corresponding to from 18 to 110 µM for RNase A and from 4 to 23 µM for HSA) and analyzed in triplicate. The resulting calibration curves (y = 31.3x (µM) + 30.4, R2 = 0.999 for RNase A; y = 339.9x (µM) ‐231.6, R2 = 0.999 for HSA) were used to quantify glycoconjugates concentration in the samples prior to GCI analysis.

### 4.8 GCI Analysis

GCI experiments were carried out on a Creoptix WAVE instrument (Malvern Panalytical, Wädenswil, Switzerland) controlled by a Creoptix WAVE control software (4.5.18 version). A 4PCH sensor chip functionalized with a high capacity polycarboxylate layer was selected. The running buffer was composed of 10 mM HEPES, pH 7.4, 150 mM NaCl, 1 mM CaCl_2_, 1 mM MgCl_2_, 0.05% Tween 20. A constant chip temperature of 25 °C was applied.

ConA immobilization was performed by amine coupling. For the activation step, a freshly prepared solution of 200 mM EDC and 50 mM NHS was injected for 420 s on the chip surface at a flow rate of 10 µL/minute. Then, a 50 µg/mL ConA solution in 10 mM sodium acetate buffer, pH 4.5 was injected twice at 10 µL/minute for 420 s on one of the chip channels. A second channel was used as a reference. Blocking of the unreacted groups was carried out by the injection of 1 M ethanolamine, pH 8.5 for 420 s at a flow rate of 10 µL/minute.

For Me‐Man kinetic analyses, a concentration range of 6.25–800 µM was used. 8 concentrations were analyzed, with a dilution factor of 2 and performing 2 replicate analyses for each concentration. Analyses were carried out at a flow rate of 50 µL/minute, with an association time of 120 s and a dissociation time of 120 s.

RNase B, RNase A, HSA, and *neo*‐glycoprotein kinetic analyses were performed at a flow rate of 10 µL/minute. The molecules were tested in serial dilutions, in a concentration range of 2.4–312.5 nM and applying a dilution factor of 2. Analytes were injected for 180 s over the two channels of the sensor chip, with a dissociation time of 180 s. After each concentration, a regeneration step was performed by injecting Me‐Man at 50 µL/minute for 120 s, with a dissociation time of 120 s. Between samples, a regeneration sequence was run, including two injections of 300 mM Me‐Man (50 µL/minute, 120 s association, and 120 s dissociation) and two injections of 1 M NaCl (50 µL/minute, 120 s association, and 120 s dissociation) alternating with running buffer injections (10 µL/minute, 180 s association, and 180 s dissociation).

Sensorgrams were processed by the Creoptix WAVE control software (4.5.18 version) using the equilibrium (Me‐Man) or kinetic (RNase B, RNase A, HSA, and *neo*‐glycoproteins) analysis evaluation tool, with a double referencing and a 1:1 interaction model.

## Supporting Information

Supporting Information for this article is given via a link at the end of the document.

## Conflict of Interest

The authors declare no conflict of interest.

## Supporting information



Supporting Information

## Data Availability

The data that support the findings of this study are available in the supplementary material of this article.
