# Supplementary material for: GCI‐Based Affinity Screening of Synthetic Oligomannosides toward Concanavalin A
Source: Chemistry. 2025 Sep 20;31(59):e02137. doi: 10.1002/chem.202502137 (PMC12548513; doi:10.1002/chem.202502137)
Supplement: Supplementary file 1 — Supporting Information [file CHEM-31-e02137-s001.docx]

**Supporting Information**

GCI-Based Affinity Screening of Synthetic Oligomannosides toward Concanavalin A

Davide Rubes^[a]^ ^§^, Sara Tengattini^[a]^ ^§^, Massimo Serra^[a]^, Teodora Bavaro^[a]^, Caterina Temporini^[a]^, He Wang^[b]^, Yongmin Zhang^[b]^, Francesca Rinaldi*^[a]^, Marco Terreni^[a]^, Enrica Calleri^[a]^

^[a]^ Department of Drug Sciences University of Pavia, Via Taramelli 12, 27100 Pavia, Italy

^[b]^ CNRS, Institut Parisien de Chimie Moléculair, UMR 8232, Sorbonne Université, 4 Place Jussieu, 75005-Paris, France

^§^These authors equally contributed to this work

*Corresponding author:

Francesca Rinaldi

E-mail: francesca.rinaldi@unipv.it

**Table of contents**

**Figure S1** ESI-MS spectra of RNase A derivatives**…....…….…………………………………………..p.S1**

**Figure S2** MALDI-ToF spectra of HSA derivatives**….…..………………………………………………..p.S2**

**Figure S3** HILIC-UV trace of RNaseB**………………...…..………………………………………………..p.S3**

**Figure S4** GCI kinetic analysis of *neo*-glycoconjugates**…..…………………………………….pp.S4-S6**

**Figure S5** GCI kinetic analysis of non-glycosylated HSA**…..………………………………….......…p.S6**

**Figure S6** NMR Spectra.**………………………………………………………………………………….pp.S7-S8**

**Figure S1.** ESI-MS spectra derived from the LC-MS analysis of RNase A conjugated to (A) Man, (B) Man(α1,6)Man, (C) Man(α1,2)Man and (D) Man(α1,6)Man(α1,2)Man. Numbers represent the incorporated sugars, determined from the detected mass.

**Figure S2.** MALDI-ToF spectra of HSA conjugated to (A) Man(α1,6)Man, (B) Man(α1,2)Man and (C) Man(α1,6)Man(α1,2)Man.

**Figure S3.** UV trace from the HILIC separation of RNase B. The five glycoforms of RNase B are chromatographically resolved and indicated in the figure by their respective glycan chains. Blue square represents N-acetylglucosamine; green circle represents mannose.

**Figure S4.** GCI kinetic analysis of *neo*-glycoconjugates: RNase A conjugated to (A) Man (**1**), (B) Man(α1,6)Man (**5**), (C) Man(α1,2)Man (**6**) and (D) Man(α1,6)Man(α1,2)Man (**10**); HSA conjugated to (E) Man(α1,6)Man (**5**), (F) Man(α1,2)Man (**6**) and (G) Man(α1,6)Man(α1,2)Man (**10**).

**Figure S5.** GCI kinetic analysis of non-glycosylated HSA, showing no interaction with the immobilized ConA.


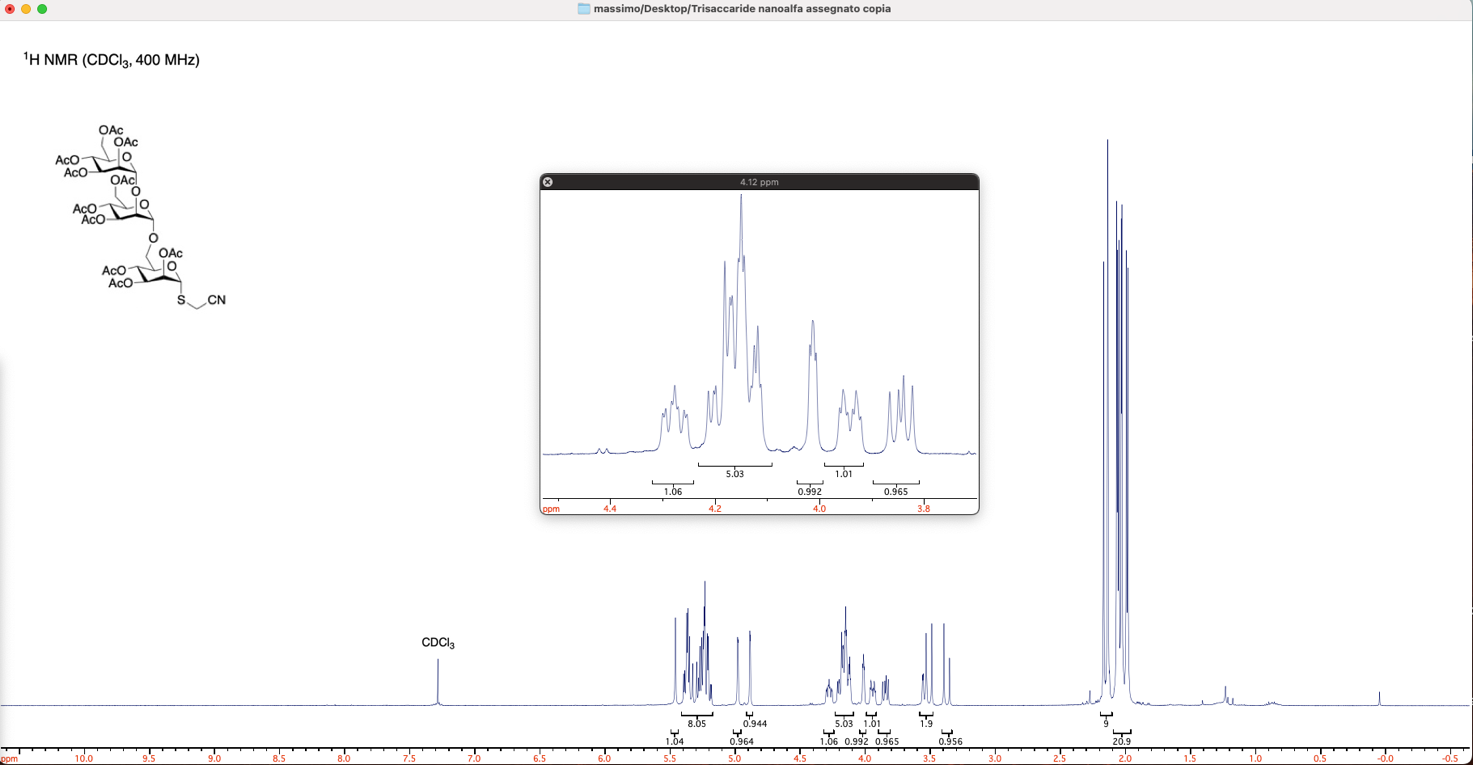


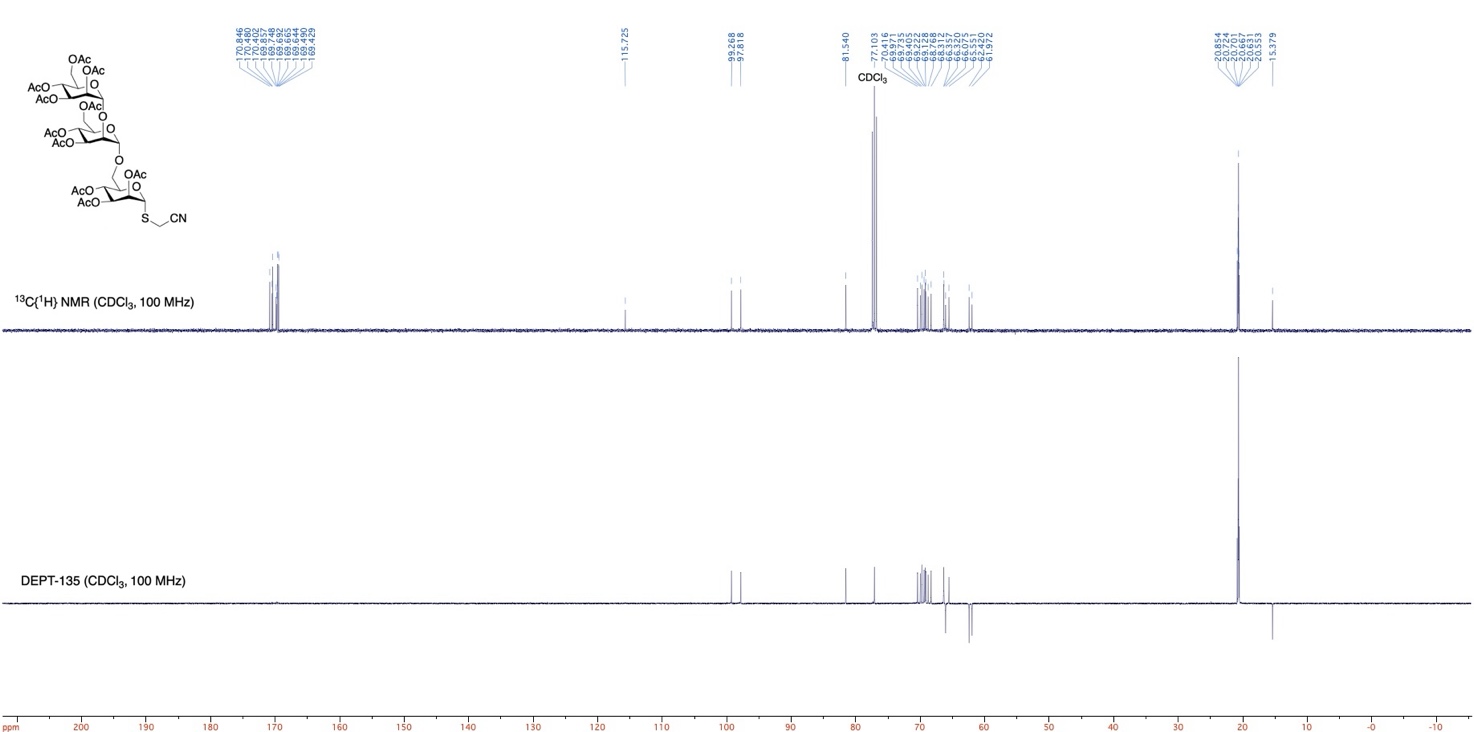


**Figure S6.** ^1^H-NMR, ^13^C{1H} NMR and DEPT-135 spectra of Man(α1,2)Man(α1,6)Man-SCH_2_CN **10**.


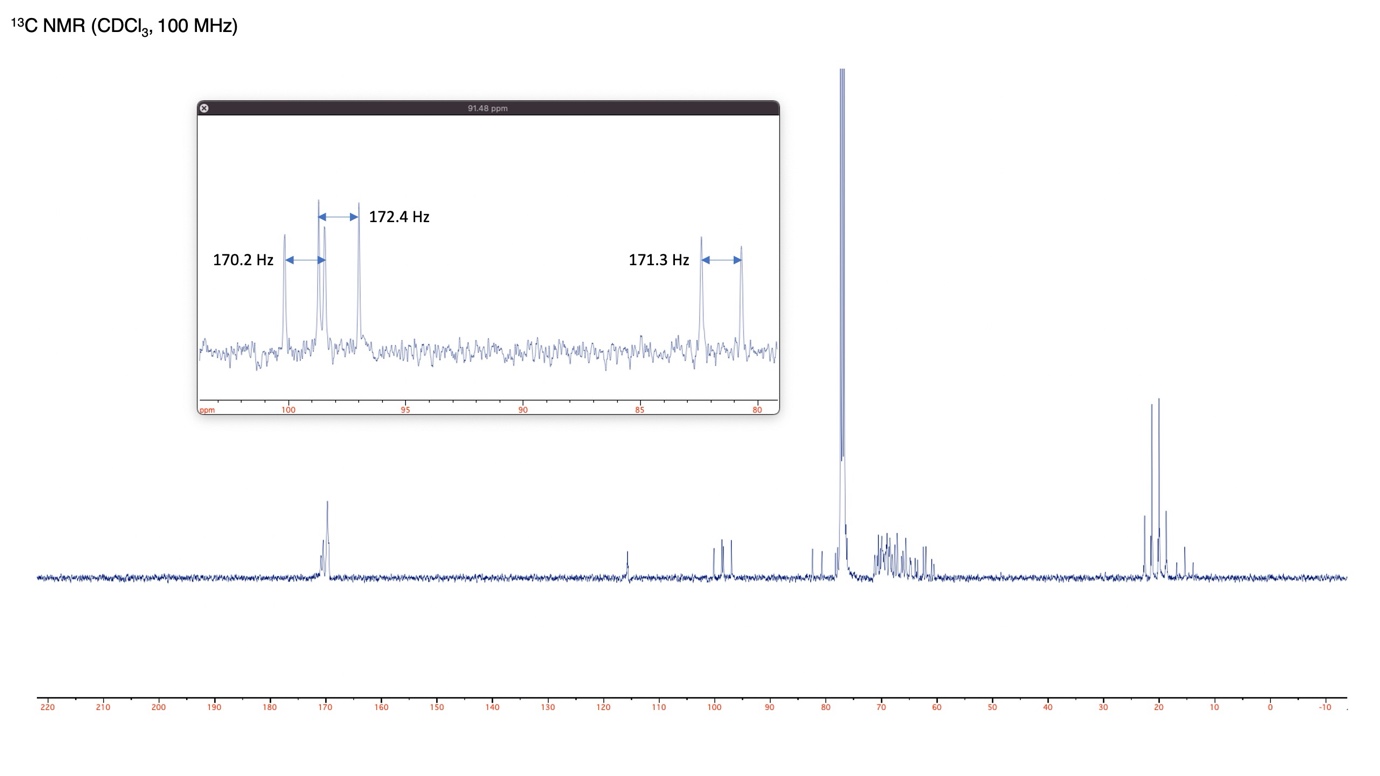
**Figure S7.** Undecoupled ^13^C NMR of Man(α1,2)Man(α1,6)Man-SCH_2_CN **10.** The ^1^J_C1,H1_ values for the anomeric carbons are shown in the box.
